# Intracranial Rosai Dorfman Disease Presented With Multiple Huge Intraventricular Masses: A Case Report

**DOI:** 10.3389/fsurg.2022.766840

**Published:** 2022-03-21

**Authors:** Elena Jamali, Guive Sharifi, Soudeh Ghafouri-Fard, Farahnaz Bidari Zerehpoosh, Mahmood Yazdanpanahi, Mohammad Taheri

**Affiliations:** ^1^Department of Pathology, Loghman Hakim Hospital, Shahid Beheshti University of Medical Sciences, Tehran, Iran; ^2^Skull Base Research Center, Loghman Hakim Hospital, Shahid Beheshti University of Medical Sciences, Tehran, Iran; ^3^Department of Medical Genetics, Shahid Beheshti University of Medical Sciences, Tehran, Iran; ^4^Department of Neurosurgery, Loghman Hakim Hospital, Shahid Beheshti University of Medical Science, Tehran, Iran; ^5^Urology and Nephrology Research Center, Shahid Beheshti University of Medical Sciences, Tehran, Iran

**Keywords:** Rosai-Dorfman disease, intraventricular, central nervous system, surgery, extranodal

## Abstract

**Introduction:**

Rosai-Dorfman disease (RDD) usually presents with painless cervical lymphadenopathy during the two first decades of life, with or without extranodal involvement. Exclusive extranodal manifestation, and especially central nervous system (CNS) involvement, is uncommon. The etiology remains unknown and definitive diagnosis is based on characteristic histopathological and immunohistochemical features showing S100(+) CD1a(−) RDD type histiocytes with emperipolesis. Most CNS cases are dural-based masses.

**Case Presentation:**

Herein, we present a case of RDD in an 8-year-old boy, who presented with multiple huge intraventricular masses.

**Conclusion:**

Regarding rare CNS involvement and lack of established evidence-based therapeutic approaches, reports of any individual case can supply further beneficial data concerning treatment approaches and long-term effectiveness of therapeutic strategies.

## Introduction

Rosai-Dorfman disease (RDD), alternatively called as sinus histiocytosis with massive lymphadenopathy (SHML), typically shows massive, painless cervical lymphadenopathy along with high body temperature, leukocytosis, high erythrocyte sedimentation rates, and polyclonal hypergammaglobulinemia during the first or second decade of life (1). In over one-fourth of the cases, RDD involves extranodal locations, usually when massive lymphadenopathy is present. However, sometimes, the extranodal involvement reflects the dominant or even exclusive manifestation of the disease. Reports have referred to the involvement of the central nervous system (CNS) as an uncommon site of presentation. The etiology is not still clear. However, there are two highly possible factors, including infection due to a virus or another microorganism and development of a subtle unclear immunologic fault (1).

Majority of CNS instances are dural-based masses, mostly involving the two sides of the dura and mimicking meningioma (2). The definitive diagnosis is based on histopathological examination and immunohistochemistry. On MRI, one or several well-defined lesions, iso-intense to the adjacent brain parenchyma, typically appear on T1-weighted images with strong and homogeneous enhancement following the administration of gadolinium. The lesions have the appearance of heterogeneous hypo- or iso-intense masses on T2-weighted images, showing similarities to the contiguous dura (3, 4).

RDD can be misdiagnosed with different histotypes of meningioma and intracranial solitary fibrous tumors (ISFTs). A retrospective study on these rare mesenchymal neoplasms has reported a recurrence rate of about 43% for solitary fibrous tumors and an approximate recurrent rate of 27% for hemangiopericytomas (5). Thus, histopathology and immunohistochemical staining can be used for differentiating between these conditions.

Diffuse lymphoplasmacytic infiltrate and histiocytes of both typical and RDD-type, showing lymphophagocytosis with no destruction of cells (emperipolesis), are characteristic histopathologic features. RDD histiocytes have bigger, more hyperchromatic nuclei compared to the other type. Moreover, they are sometimes multinucleated with plentiful light pink cytoplasm, and show immunoreactivity for CD68 and S100 but they are negative for CD1a on immunohistochemistry (2).

In several instances, RDD histiocytes undergo quick and full spontaneous resolution. In other cases, particularly those which show extensive extranodal involvement, a long clinical course may be experienced for several years or even decades. Therapy does not usually affect RDD, even though there have been several instances of successful chemotherapy (1). It is recommended to treat RDD affecting the lymph nodes and extranodal sites out of CNS when the patient has symptomatic lesions or masses preventing the function of vital organs (4). Complete surgical resection, if feasible, is the preferential therapeutic option that serves diagnostic objectives and also improves neurologic symptoms of patients experiencing CNS involvement. When neurologic symptoms persist or there are lesions around vital structures, the implementation of adjuvant therapies is recommended, together with localized radiotherapy (4). Some case reports have suggested a therapeutic trial of steroids in systemic RDD, which has constitutional symptoms (6, 7), or in the case of CNS involvement (6–8). Different chemotherapy regimens have been used for the treatment of progressive systemic RDD, but there is no established evidence-based therapeutic approach for CNS involvement due to the rarity of this entity. Herein, we present a case of RDD presented with multiple huge intraventricular masses in a boy aged 8 years. Based on the rarity of this situation, it is important to consider RDD in the differential diagnosis of cases with similar presentations and choose the appropriate management options available for these challenging scenarios.

## Case Characterization

**Figure 1:**
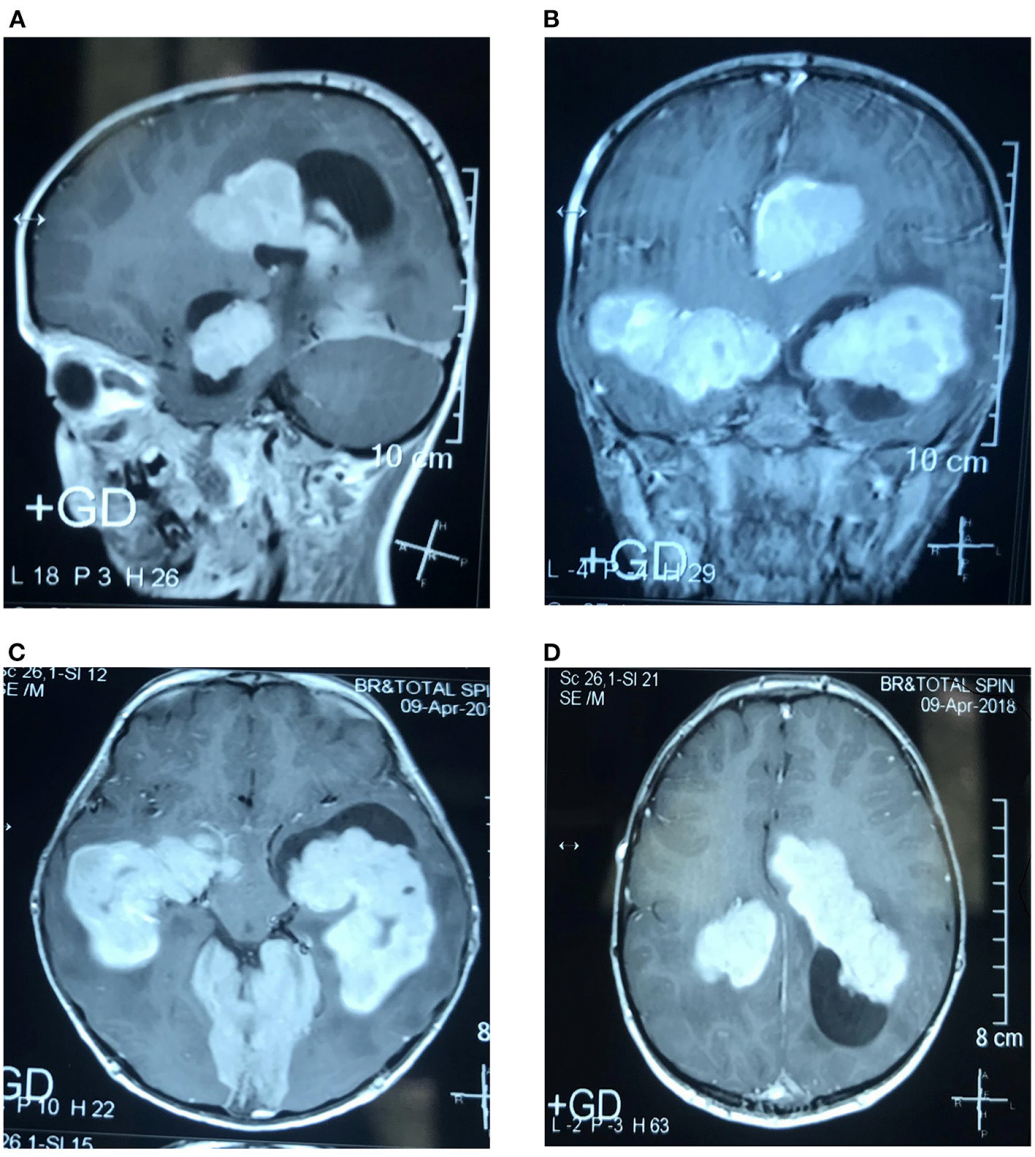
Sagittal **(A)**, coronal **(B)**, and axial **(C,D)**. T1-weighted (T1W) contrast-enhanced MRI show multiple, homogeneously enhancing intraventricular masses.

**Figure 2:**
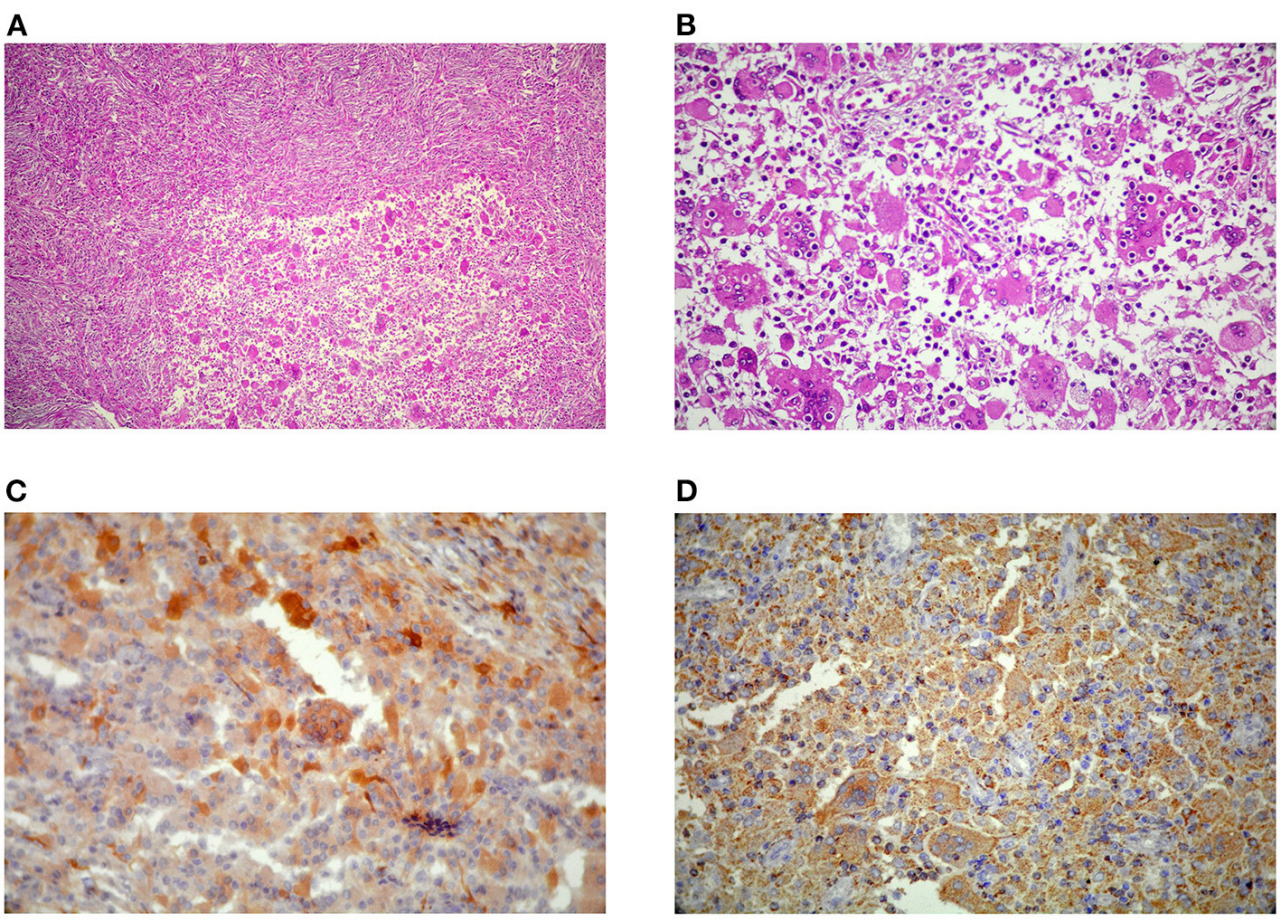
**(A)** Diffuse proliferation of Rosai-Dorfman disease (RDD) histiocytes with characteristic emperipolesis **(B)** in a densely fibrotic stroma admixed with lymphoplasmacytic infiltrate (H&E, x100, and x400). Large histiocytes immunoreactive for S100 **(C)** and CD68 **(D)** and immunonegative for CD1a (not shown here).

A boy aged 8 years, suffering from multiple intraventricular huge masses resulting in hydrocephalus, was referred to the neurosurgery ward of the Loghman Hakim Hospital. Written informed consent forms were signed by the parents. The study protocol was approved by the Ethical Committee of Shahid Beheshti University of Medical Science, and all methods were performed in accordance with the relevant guidelines and regulations (IR.SBMU.RETECH.REC.1400.385). He had initially presented with fever, dizziness, and bitemporal headache about 1.5 years ago and was admitted in a pediatric center with an impression of meningitis. However, lumbar puncture was not performed at that time because of brain edema, bilateral papilledema as well as evidence of choroid plexus hypertrophy and abnormal extra-intracranial vascular connections. He had normal chest radiography and abdominopelvic sonography. He had been discharged in a relatively good condition after antibiotic therapy. On this admission, he presented with nausea, vomiting, and ataxia. On examination, no sign of adenopathy or organomegaly was evident. Brain MRI revealed large homogeneously enhancing bilateral ventricular lesions that seemed to configure the shape of the whole ventricles (Figure 1). As the main clinical complaint was the constellation of high intracranial pressure (ICP) symptoms, we were obliged to do effective surgical debulking. Given that no cerebrospinal fluid (CSF)-filled space was present due to huge tumoral propagation, the only solution was tumor removal. Ruling out some chemo radiosensitive tumors such as choroid plexus carcinoma and lymphoma, which may obviate surgery as the main treatment modality, we proceeded for intraoperative pathology consultation by frozen section. At first operation after a large frontotemporal craniotomy, the large ventricular lesion was approached by middle temporal gyrus cortectomy. The obtained tumor samples were evaluated and showed no highly malignant tumor or hematologic malignancy, which persuaded us to do total tumor resection. The tumor resection was done in a manner of resection of a huge trigonal meningioma. One week later, we went through the same approach but from the right intraparietal sulcus, removing the huge lateral ventricle compartment and evacuating the third ventricular fragment through the foramen of Monro. There was no external ventricular drainage, and post-operative courses were uneventful. Post-operative imaging showed ventricular dilation as an encephalomalacia characteristic and no high ICP was detected by examining the post-operative CT scan images and clinical evaluation (Figures 2A,B). Neurologic examination showed mutism without significant motor deficit.

**Figure 3:**
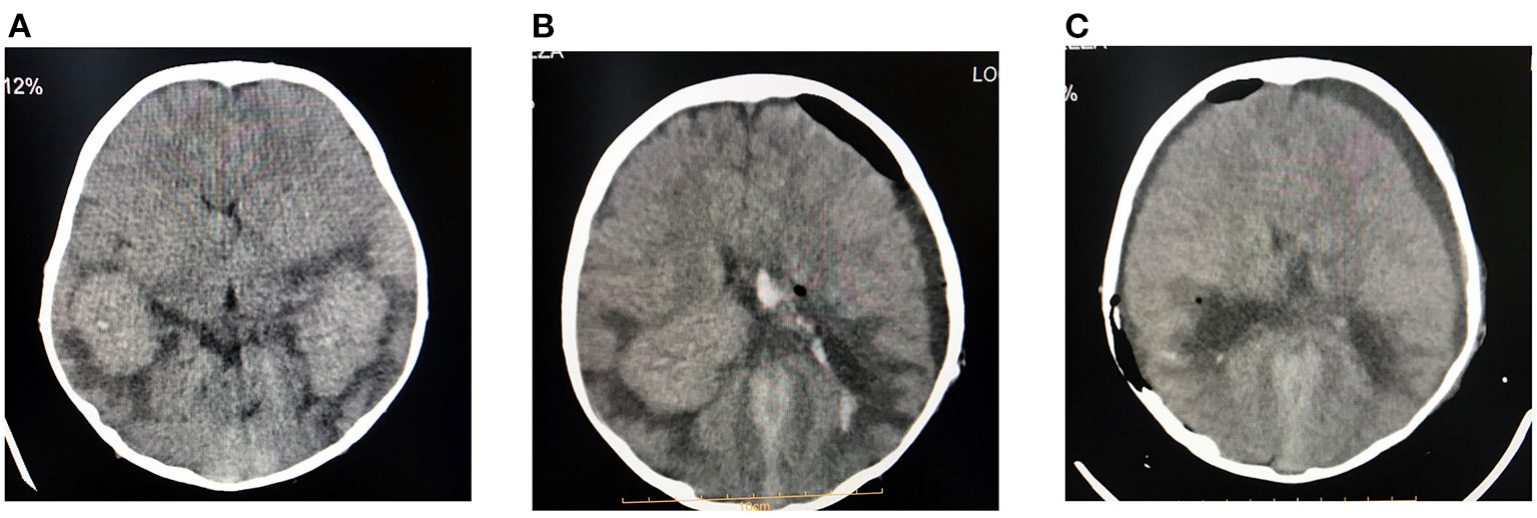
Preoperative **(A)**, first post-operative **(B)**, and second post-operative **(C)** computed tomography (CT) images. Total tumor resection is illustrated.

Histopathologic examination revealed diffuse proliferation of histiocytic cells, some with abundant pale eosinophilic cytoplasm and characteristic emperipolesis in a densely fibrotic stroma admixed with a variable amount of lymphoplasmacytic infiltrate (Figures 2C,D). Staining showed negative results for organisms. Large histiocytes showed positive results for CD68 and S100 on immunostatining, while the result for CD1a was negative (Figure 3), thereby establishing the RDD diagnosis. The patient's mother declared about a thigh skin lesion excised when the boy was 4 years old. The corresponding pathology report was consistent with a benign fibrohistiocytic tumor. After all, with the diagnosis of RDD with predominant CNS involvement, the patient underwent localized radiotherapy (total dose of 45Gy divided in single doses of 108 Gy) and chemotherapy following a short interval. Adjuvant systemic chemotherapy with prednisolone and methotrexate continued for 12 weeks without improvement in neurologic deficits. The patient remained stable at 12 months follow-up and had no recurrence of intracranial lesions on control imaging.

## Discussion

Rosai-Dorfman disease can involve almost any organ system and affect any age group. The CNS involvement is uncommon, occurring in around 5% of cases (9). Nevertheless, involvement of the CNS indicates no worse prognosis. Those suffering such a complication usually belong to a higher age range, having a median age of 39 years, with males showing nearly two times higher likelihood of being affected. Given that most CNS RDD cases appear to be dural-based, the disease is commonly diagnosed as meningioma in preoperative assessments. It may also mimic subdural hemorrhage, ependymomas, and dysplastic gangliocytoma of the cerebellum. There are rare presentations of RDD as intraventricular masses, similar to the case presented here, or dural, venous sinuses, and bone lesions (3, 10). Nearly 90% of CNS RDD cases are intracranial lesions, however, it is possible to observe dural-based masses in the vertebral canal usually causing compression of the spinal cord and subsequent paraplegia (9, 11–13). The available literature has described some cases of intraventricular presentation (12, 14) in adult patients, and our case demonstrates multiple intraventricular masses in a child. Another study has reported an RDD patient who had two isolated lesions in the CNS being removed sequentially in two operations. Patient experienced no seizure or recurrence during the follow-up period (15). Moreover, there is a report of a 2-year-10-month-old girl with RDD, who had fever, vomiting, and a history of muscular weakness, having recovered wholly. In her brain MRI, she had an axial enhancing lesion with ventricular spreading. Symptoms had been amended by steroid therapy. Moreover, the occipital intraventricular lesion had been partially removed (16). Finally, a recent study has reported a 30-year-old man, a case of RDD presented as an isolated right cerebellar peduncle lesion bulging into the fourth ventricle (17).

Iso-intensity or hyper-intensity and homogeneity of RDD lesions of the CNS can be evident on computed tomography (CT or CAT), enhanced by contrast administration, but with no calcifications. On the other hand, MRI shows the homogeneity and isointensity of RDD lesions on T1-weighted images (T1WI) while indicating heterogeneity of RDD lesions with isointense and hypointense areas on T2-weighted images (T2WI). Gadolinium administration enhances the lesions homogeneously, usually showing a dural tail. Both CT scan and MRI methods usually indicate perilesional edema (18, 19).

There are similarities between the RDD histopathological characteristics in extranodal manifestations and nodal involvement. The only difference is in more distinct fibrosis and less obvious emperipolesis in extranodal sites (1). Similarly, in our case, the excessive fibrosis and collagenization obscured the background cells and made the frozen section diagnosis to be deferred.

Rosai-Dorfman disease of the CNS is preferably managed by surgical resection that relieves neurologic symptoms. Adjuvant therapies are taken into account when neurological symptoms persist or lesions appear around vital structures. There are different therapies, including fractionated radiotherapy, stereotactic radiotherapy, corticosteroids, and chemotherapy, but more investigations need to be performed to find the optimal management of the disease (6). A systematic review of the literature to assess the impact of stereotactic radiosurgery in intracranial histiocytosis has been conducted on seven studies including two cases of RDD. Authors have stated that there is insufficient data for making conclusions regarding the role of stereotactic radiosurgery in these lesions (18). The application of various systemic agents, such as vinca alkaloids, anthracyclines, alkylating agents, methotrexate, interferon-α, and Imatinib, has been partially successful in RDD (6, 9). It may be possible to set up beneficial targeted therapies with more knowledge on the exact etiology and pathogenesis of RDD. The chemotherapeutic regimen in our case started initially with prednisolone and continued with methotrexate for 12 weeks. The patient had no recurrence in a one-year follow-up, but his post-operative neurological defects remained persistent.

Chemotheraputic modality is used to shrink space occupying lesions alongside other treatment modalities. Rarely, RDD can be presented as two isolated lesions that originated from a single clone (15). Similarly, adjuvant radioenhancers have been used to optimize radiosurgery protocols in the comprehensive management of residual/recurrent disease. In fact, radioenhancers can promote the toxic effects of radiation in tumor cells while decreasing the toxicity to adjacent healthy tissues (20).

## Conclusion

Rosai-Dorfman disease of the CNS can be defined as an uncommon presentation, which requires consideration in differential diagnosis of not only dural-based but also unusual intraventricular brain lesions, especially in children. Definitive diagnosis is according to the histopathology and immunohistochemistry. Surgical resection seems to be the most effective therapy, and given that the disease is rare, evidences obtained from any individual case studies can provide further important information regarding the treatment approaches and the long-term effectiveness of treatment strategies for relapse/incompletely resected lesions.

## Limitation of the Study

Our study had a limitation regarding lack of long term follow-up. Further assessment of additional similar cases is also required for estimation of recurrence rate and efficacy of therapeutic modalities.

## Data Availability Statement

The raw data supporting the conclusions of this article will be made available by the authors, without undue reservation.

## Ethics Statement

The studies involving human participants were reviewed and approved by Shahid Beheshti University of Medical Sciences. The patients/participants provided their written informed consent to participate in this study.

## Author Contributions

EJ and GS wrote the draft and revised it. MT, FB, and MY collected the data and performed the clinical assessment. All authors read and approved submitted version.

## Conflict of Interest

The authors declare that the research was conducted in the absence of any commercial or financial relationships that could be construed as a potential conflict of interest.

## Publisher's Note

All claims expressed in this article are solely those of the authors and do not necessarily represent those of their affiliated organizations, or those of the publisher, the editors and the reviewers. Any product that may be evaluated in this article, or claim that may be made by its manufacturer, is not guaranteed or endorsed by the publisher.

## References

[1] Rosai J. Rosai and Ackerman's Surgical Pathology e-book. Elsevier Health Sciences 2011.

[2] Kleinschmidt-DeMasters BK Tihan T Rodriguez F. Diagnostic Pathology: Neuropathology. Elsevier Health Sciences (2016).

[3] Adeleye AO Amir G Fraifeld S Shoshan Y Umansky F Spektor S. Diagnosis and management of Rosai–Dorfman disease involving the central nervous system. Neurol Res. (2010) 32:572–8. 10.1179/016164109X12608733393836 20350368

[4] Sandoval-Sus JD Sandoval-Leon AC Chapman JR Velazquez-Vega J Borja MJ Rosenberg S. Rosai-Dorfman disease of the central nervous system: report of 6 cases and review of the literature. Medicine. (2014) 93:165–75. 10.1097/MD.0000000000000030 24797172 PMC4632912

[5] Gubian A Ganau M Cebula H Todeschi J Scibilia A Noel G. Intracranial solitary fibrous tumors: a heterogeneous entity with an uncertain clinical behavior. World Neurosurgery. (2019) 126:e48–56. 10.1016/j.wneu.2019.01.142 30716501

[6] Cooper SL Jenrette MJ. Rosai-Dorfman disease: management of CNS and systemic involvement. Clin Adv Hematol Oncol. (2012) 10:199–202. 22402430

[7] Hadjipanayis CG Bejjani G Wiley C Hasegawa T Maddock M Kondziolka D. Intracranial Rosai—Dorfman disease treated with microsurgical resection and stereotactic radiosurgery: case report. J Neurosurg. (2003) 98:165–8. 10.3171/jns.2003.98.1.0165 12546366

[8] Johnson MD Powell SZ Boyer PJ Weil RJ Moots PL. Dural lesions mimicking meningiomas. Hum Pathol. (2002) 33:1211–26. 10.1053/hupa.2002.129200 12514791

[9] Warrier R Chauhan MA Jewan Y Bansal S Craver R. Rosai-Dorfman disease with central nervous system involvement. Age. (2012) 6:17. 22402429

[10] Morandi X Godey B Riffaud L Heresbach N Brassier G. Isolated Rosai—Dorfman disease of the fourth ventricle: case illustration. J Neurosurg. (2000) 92:890. 10.3171/jns.2000.92.5.0890 10794310

[11] Wu M Anderson AE Kahn LB. A report of intracranial Rosai-Dorfman disease with literature review. Ann Diagn Pathol. (2001) 5:96–102. 10.1053/adpa.2001.23027 11294995

[12] Andriko J-AW Morrison A Colegial C Davis BJ Jones RV. Rosai-Dorfman disease isolated to the central nervous system: a report of 11 cases. Modern Pathology. (2001) 14:172–8. 10.1038/modpathol.3880278 11266522

[13] Qin G Ye J Lan S Liang Y Xu P Tang X. Rosai-Dorfman disease with spinal and multiple intracranial involvement: a case report and literature review. Br J Neurosurg. (2019) 1–5. 10.1080/02688697.2019.1567681 30773931

[14] Patwardhan PP Goel NA. Isolated intraventricular Rosai–Dorfman disease. Asian J Neurosurg. (2018) 13:1285. 10.4103/ajns.AJNS_134_18 30459919 PMC6208248

[15] Jin H Yu Z Tian T Shen G Chen W Fan M. Rosai-Dorfman disease in the central nervous system with two isolated lesions originated from a single clone: a case report. BMC Neurol. (2021) 21:352. 10.1186/s12883-021-02379-2 34517832 PMC8436543

[16] Lüdemann W Banan R Samii A Koutzoglou M Di Rocco C. Cerebral Rosai–Dorfman disease. Child's Nervous System. (2015) 31:529–32. 10.1007/s00381-015-2629-2 25686890

[17] Friconnet G Duchesne M Gueye M Caire F Mounayer C Emile J-F. Isolated cerebral Rosai–Dorfman disease presenting as a sole mass protruding into the fourth ventricle: a case report. Radiol Case Rep. (2021) 16:1613–7. 10.1016/j.radcr.2021.04.021 33995752 PMC8105597

[18] Zhu H Qiu L-H Dou Y-F Wu J-S Zhong P Jiang C-C. Imaging characteristics of Rosai-Dorfman disease in the central nervous system. Eur J Radiol. (2012) 81:1265–72. 10.1016/j.ejrad.2011.03.006 21440399

[19] Raslan OA Schellingerhout D Fuller GN Ketonen LM. Rosai-Dorfman disease in neuroradiology: imaging findings in a series of 10 patients. Am J Roentgenol. (2011) 196:W187–93. 10.2214/AJR.10.4778 21257861

[20] Ganau M Foroni RI Gerosa M Ricciardi GK Longhi M Nicolato A. Radiosurgical options in neuro-oncology: a review on current tenets and future opportunities. Part II: adjuvant radiobiological tools. Tumori J. (2015) 101:57–63. 10.5301/tj.5000215 25702646

